# Round the World

**Published:** 1914-01-31

**Authors:** 


### ROUND THE WORLD.

### Fellow-Workers and their Doings.

**[The Editor Invites Early Information and Contributions Marked "Fellow-Workers."**

Professor G. Sims Woodhead, M.D., F.R.C.P., who has just been appointed honorary consulting pathologist to the Royal National Hospital for Consumption at Ventnor, was, of course, one of the members of the Royal Commission on Tuberculosis appointed in 1902. Professor Sims Woodhead's work at Cambridge, where he has been Professor of Pathology since 1889, has been connected with most of the important developments in this branch which have taken place during the last fifteen years. An old Edinburgh student, the distinguished pathologist was for many years President of the Edinburgh University Athletic Club.

---

Dr. F. W. Andrewes, director of the pathological department at St. Bartholomew's Hospital, is carrying out some very interesting researches there in an attempt to estimate the amount of calcium salts present in the arteries. Much attention has, of course, been drawn to the importance of calcium salts in metabolism by the recent observations of Dr. Blair Bell at Liverpool. Before the value of the calcium-content of any blood-vessel can be judged in dealing with the problem of calcareous degeneration, it is necessary to settle as far as possible the relative quantities of calcium present at different periods of life. It is understood that the method being pursued at "Bart.'s" consists in obtaining arteries in quantity from the *post-mortem* room, and subjecting the mass to incineration after it has been thoroughly dried; the resulting ash being then analysed in the ordinary way.

---

Dr. Emily E. Flemming, M.D., who has been appointed part-lecturer in medicine at the Royal Free Hospital (London School of Medicine for Women), was formerly medical registrar and demonstrator of anatomy at these affiliated institutions. Dr. Flemming is a member of the staff of the New Hospital for Women, Euston Road.

---

Mr. Malcolm Hepburn, M.D., F.R.C.S., who has been appointed part-lecturer in ophthalmology at the same school, has been ophthalmic surgeon to the hospital for some time; he is also assistant surgeon to the Royal London Ophthalmic Hospital, and was formerly on the staff of the Central London Ophthalmic Hospital, as well as surgeon to the eye department (out-patients) at the Hampstead General Hospital.

---

At Guy's Hospital the competition for the Newland-Pedley Gold Medal in practical dentistry (1913) has resulted in the award being made to Mr. A. E. Lowein; whilst a certificate of distinction has been given to Mr. R. J. Ryland.

---

Dr. R. Fortescue Fox, M.D., for many years a student of balneology, especially in connection with Strathpeffer, has now brought out a book on medical hydrology. The treatment by waters and baths is a subject that is as yet but little understood by practitioners in general, and further authoritative statements from the scientific side are badly needed.

---

Another subject of which much the same might be said is radium therapy, in connection with which Mr. N. S. Finzi, M.B., M.R.C.S., whose name is well known in regard to original observation in this country as to the effect of radium on cancers of the throat and surface of the body, has now embodied his experiences in a volume entitled "Radium Therapeutics." The author has had the great advantage in his investigations of the large clinic at St. Bartholomew's Hospital, where he is chief assistant in the *x*-ray department.

---

Dr. H. B. Carlill, M.D., who went from Guy's to be medical registrar at the Westminster Hospital, and was at one time assistant physician to the Belgrave Hospital for Children, has transferred some of his hospital interests to the south-eastern district, having been appointed physician to the Miller General Hospital at Greenwich. Dr. Carlill worked in the Educational Department (inspection of school children) of the London County Council, and two or three years ago visited the laboratories of Professor Kitasato in Japan.

---

Mr. Henry S. Souttar, F.R.C.S., is fortunate in the possession of a gift for mechanical inventions. His abilities in this direction were well exemplified by the gastroscope which he designed a few years ago, and which was admitted on all sides to be a marvellously ingenious instrument. Mr. Souttar's latest efforts have been directed towards the improvement of surgical operating tables, and it seems likely that he will soon be able to place before the profession a more generally adaptable table than has yet been constructed. At the West London Hospital Mr. Souttar has done much to popularise the use of stovaine as an anæsthetic in operations on the abdomen and lower limbs.

---

Dr. W. J. Pearson succeeds Dr. H. L. Tasker as resident medical officer at University College Hospital, his predecessor having embarked on medical practice in the London area. The *University College Hospital Magazine* is our authority for stating that Dr. Pearson holds the record for the longest drive ever made on the Cromer golf links.

